# *Onosma armenum* Extract Loaded Mesoporous Silica Nanoparticles for the Prevention of Biofilm‐Associated Infections

**DOI:** 10.1002/open.202500554

**Published:** 2026-03-29

**Authors:** Sevim Feyza Erdoğmuş, Nilay Isitez, Emre Burak Ertuş, Cengiz Sarikurkcu

**Affiliations:** ^1^ Department of Basic Pharmaceutical Sciences Faculty of Pharmacy Afyonkarahisar Health Sciences University Afyonkarahisar Turkey; ^2^ Department of Mechanical Engineering KTO Karatay University Konya Turkiye

**Keywords:** antibiofilm, nanoparticles, *Onosma armenum*, phenolic content, *Staphylococcus aureus*

## Abstract

In this study, bioactive mesoporous silica nanoparticles (MSNPs) loaded with *Onosma armenum* extract were developed to evaluate their antibiofilm activity against biofilm‐forming *Staphylococcus aureus* ATCC 25923. The phytochemical content of the extract was analyzed via liquid chromatography–electrospray ionization tandem mass spectrometry. MSNPs were synthesized using modified MCM‐41 method and characterized by N_2_ adsorption–desorption tests, scanning and transmission electron microscopy, X‐ray diffraction, and Fourier‐transform infrared spectroscopy. The antibiofilm activity was assessed using 3‐(4,5‐dimethylthiazol‐2‐yl)−2,5‐diphenyltetrazolium bromide reduction assay. The most abundant compounds were hesperidin (14201 µg/g extract), chlorogenic acid (4390 µg/g extract), and rosmarinic acid (830 µg/g extract). Biofilm inhibition rates were determined as 31.97 ± 0.68% (extract), 72.78 ± 0.79% (MSNPs), and 76.49 ± 0.39% (extract‐loaded nanoparticles) at 2× MIC concentration, while biofilm eradication rates were 23.66 ± 0.44%, 36.33 ± 0.40%), and 42.27 ± 0.44%, respectively. Scanning electron microscopy analyses revealed the morphological effects of nanoparticles on biofilm structures. The nanoparticles exhibited effective loading and controlled release of plant extract. The extract‐loaded MSNPs demonstrated significant antibiofilm activity and controlled release in acidic environments, indicating potential for targeted therapeutic applications. The synergy between phytochemicals and nanocarriers supports their use as promising agents against biofilm‐associated infections.

## Graphical abstract

Bioactive mesoporous silica nanoparticles (MSNPs) loaded with *O. armenum* extract were developed to evaluate their antibiofilm activity against biofilm‐forming *Staphylococcus aureus* ATCC 25923. The phytochemical content of the extract was analyzed via liquid chromatography–electrospray ionization tandem mass spectrometry. MSNPs were synthesized using a modified MCM‐41 method and characterized by N_2_ adsorption–desorption tests, scanning and transmission electron microscopy, X‐ray diffraction, and Fourier‐transform infrared spectroscopy. The antibiofilm activity was assessed using the MTT reduction assay.

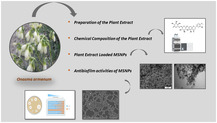

## 1 Introduction

Antibiotic resistance represents a significant challenge in the treatment of infections, with biofilm formation playing a critical role in this resistance. Consequently, there is an urgent need for novel biofilm‐inhibiting compounds to combat persistent pathogens resistant to antibiotics [1]. Biofilms are defined as microbial communities that adhere to surfaces and are embedded within a self‐produced, gel‐like polymeric matrix. The extracellular matrix, composed of exopolysaccharides and proteins, serves as an essential attachment surface for biofilm formation [2]. Biofilms consist of ≈97% water, along with 2–5% microorganisms, 1–2% polysaccharides, 1–2% proteins, 1–2% DNA, and various ions. The extracellular matrix provides a protective barrier against harsh environmental conditions and antimicrobial agents, resulting in a significant increase in the resistance of bacteria within biofilms compared to their planktonic counterparts [3]. Studies have demonstrated that biofilm‐forming bacteria can exhibit up to a 100‐ to 10,000‐fold increase in antibiotic resistance compared to planktonic bacteria. Furthermore, some bacteria that are initially susceptible to antimicrobial agents can develop resistance once they initiate biofilm formation; however, they may regain susceptibility once they detach from the biofilm [4]. Biofilm formation is a multistep and complex process involving genes that play crucial roles in cell physiology, influencing adhesion, quorum sensing, cell wall structure, metabolism, stress responses, and plasmid attachment [5, 6]. The increased resistance of biofilm‐embedded bacteria to antibiotics is largely attributed to the enhanced transfer of antibiotic resistance genes among bacterial cells within the biofilm [7].

*Staphylococcus aureus* is a significant pathogen responsible for infections in both humans and animals. As a commensal of the skin and mucosal surfaces, *S. aureus* causes various skin and soft tissue infections. Moreover, due to its virulence factors, it can lead to severe infections such as bacteremia, toxic shock syndrome, osteomyelitis, and meningitis. The rapid emergence and spread of multidrug‐resistant strains, particularly methicillin‐resistant *S. aureus*, pose a major challenge in the treatment of these infections [8].

In the context of the rising prevalence of antibiotic‐resistant bacteria, there is an urgent need for novel antibacterial therapies. Nanostructured materials represent a promising avenue for the development of innovative antibacterial drug delivery systems. Among these, mesoporous silica nanoparticles (MSNPs) have garnered significant attention due to their thermal, chemical, and mechanical properties, which make them ideal drug carriers. In addition to these properties, MSNPs offer distinct advantages over other drug delivery systems, including ease of synthesis and cost‐effectiveness. The morphology, pore size, pore volume, and particle size of MSNPs can be tailored by modifying synthesis parameters. Recent research on MSNPs as drug carriers for the treatment of various diseases has demonstrated their versatility in loading small molecules, proteins, siRNAs, and similar macromolecules [9]. Porous silicas constitute a substantial family of inorganic materials, characterized by open‐pore structures and elevated surface areas.

Plants have been found to be abundant in antimicrobial and other pharmacological compounds [10, 11]. Compounds such as diterpenoids [12], oleic acid [13], ellagic acid [14], esculetin and fisetin [15], 1,2,3,4,6‐penta‐O‐galloyl‐ß‐D‐glucopyranose [16], and tannic acid [17] have been reported to inhibit biofilm formation by *S. aureus*. Controlled release systems containing natural bioactive compounds with antimicrobial activity hold great potential for infection treatment.

The Boraginaceae family is generally found in tropical and subtropical climates. The genus *Onosma* L., belonging to the Boraginaceae family, occurs in tropical, subtropical, and temperate regions worldwide. Türkiye is the richest Asian country in terms of *Onosma* species (95 species), followed by China and Pakistan, with 29 and 8 species, respectively. Although earlier reports indicated ≈200 species worldwide, ongoing systematic botanical studies suggest that the number of *Onosma* species is increasing, now estimated at around 230 species. The medicinal and industrial significance of *Onosma* species has led researchers to conduct extensive studies on their phytochemical and biological activities in recent years [18, 19]. In traditional medicine, *Onosma* species are widely used to treat ailments such as rheumatism, palpitations, blood disorders, bronchitis, leucoderma, fever, wounds, burns, and other conditions. The roots of the plant are used to control fever, relieve pain, treat wounds, and combat infectious diseases and stings. It has also been reported to be applied topically for skin disorder treatment [20]. *Onosma* species are among the plant species whose biological activities are still being explored. Although research on these species remains limited, the increasing number of studies highlights their significance. *Onosma* species characteristically contain phenolic compounds, alkaloids, and naphthoquinones. Additionally, alkanins and shikonins found in *Onosma* species, also present in other Boraginaceae family members, exhibit biological activities such as wound healing, analgesic, anti‐inflammatory, and antimicrobial effects [21]. Some studies on the antioxidant activities of *Onosma* species have demonstrated their strong antioxidant potential [22]. Twenty‐seven compounds were identified in *Onosma armenum* extract using liquid chromatography–electrospray ionization tandem mass spectrometry (LC‐ESI‐MS/MS), with chlorogenic acid, apigenin‐7‐glucoside, luteolin‐7‐glucoside, and hesperidin identified as major compounds. The study highlighted *O. armenum* as a valuable source for pharmacological applications [20].

In this study, bioactive MSNPs loaded with *O. armenum* extract were developed for the first time and their potential for the treatment of biofilm‐associated infections was demonstrated.

## 2 Materials and Methods

### 2.1 Plant Collection and Extraction

*O. armenum* was collected during its flowering period from Kemer/Antalya, Türkiye (15 m a.s.l., 36°31′54.12″ N, 30°32′1.32″ E), and deposited under Herbarium number OC.5044. The aerial parts of the plant were ground into powder. Ultrasonic extraction method was used for the preparation of *O. armenum* ethanol‐distilled water (1:1) extract (Ons) [23]. Each 30 g of powdered sample was placed in 400 mL of solvent and subjected to extraction by ultrasonication at 35–40°C for 1 h. The extract was filtered through Whatman No:1 filter paper and then the solvents used were evaporated in a rotary evaporator (Heidolph) under 40°C and at low pressure. After evaporation, the plant extracts were dried and concentrated at −50°C under reduced pressure in a lyophiliser to remove the last liquid residues. The powdered Ons obtained after lyophilisation were stored at +4°C until use.

### 2.2 Determination of the Phenolic Compositions of the Extracts

The total phenolic and flavonoid contents of the extracts were measured spectroscopically [24, 25], whereas the detailed phytochemical composition was analyzed by liquid chromatography–electrospray ionization tandem mass spectrometry (LC–ESI–MS/MS) using a previously validated method [26], with the analytical parameters provided in Tables 1 and 2 of the supplementary material.

**TABLE 1:** Total phenolic and flavonolic content of *O. armenum* extract.

Tests	Concentrations
Total flavonoid compound (mg REs/g extract)	27.22 ± 0.09
Total phenolic compound (mg GAEs/g extract)	75.00 ± 0.59

REs and GAEs: Rutin and gallic acide equivalents, respectively. Values are expressed as mean ± SD (*n* = 3).

**TABLE 2:** Concentration of selected phenolic compounds in the extract from *O. armenum*.

Analytes	**Concentration** **(µg/g extract)**	Analytes	**Concentration** **(µg/g extract)**
Gallic acid	0.31 ± 0.01	Sinapic acid	nd
Pyrocatechol	393±15	2‐hydroxycinnamic acid	nd
2,5‐dihydroxybenzoic acid	468±4	p‐Coumaric acid	469 ± 2
Protocatechuic acid	498±1	Ferulic acid	90.9 ± 2.3
3,4‐dihydroxyphenylacetic acid	nd	Luteolin 7‐glucoside	2.7 ± 0.2
(+)‐Catechin	nd	Hesperidin	14 201 ± 491
Chlorogenic acid	4390±130	Hyperoside	242 ± 6
3‐hydroxybenzoic acid	556±1	Rosmarinic acid	830 ± 12
4‐hydroxybenzoic acid	515±17	Apigenin 7‐glucoside	3.53 ± 0.21
(−)‐Epicatechin	nd	Pinoresinol	nd
Caffeic acid	134±1	Eriodictyol	nd
Syringic acid	12.1±0.2	Quercetin	nd
Vanillin	nd	Luteolin	nd
Verbascoside	nd	Kaempferol	nd
Taxifolin	nd	Apigenin	nd

Values are expressed as mean ± SD (*n* = 3). nd: not detected.

### 2.3 MSNPs Synthesis

MSNPs were synthesized using a modified method based on MCM‐41synsthesis. Initially, 0.6 g (0.0015 mol) of Cetyltrimethylammonium Bromide (Sigma–Aldrich, AR ≥98%) was dissolved in 50 mL of deionized water. Subsequently, 0.6 mL of a 25% ammonia (NH_3_, Sigma–Aldrich) solution was added, followed by the addition of 4 mL of Tetraethyl orthosilicate (≥99.0% GC, Sigma–Aldrich) at room temperature as the silica source. The solution was stirred at 60°C for 90 min, after which the white precipitate was collected via vacuum filtration on a Whatman 60 filter paper and washed with deionized water and ethanol in turns. The collected material was then dried at 120°C for 2 h, followed by calcination at 550°C for 4 h to completely remove surfactants and reaction byproducts, resulting in the final MSNPs. To functionalize the MSNP with amine groups, the particles were treated with a 1/5 (v/v) solution of 3‐(2‐aminoethylamino) propyl] trimethoxysilane (Sigma–Aldrich, ≥80%)/anhydrous toluene (APTES, Sigma–Aldrich, 99.8%), under reflux with magnetic stirring for 24 h at 110°C. The APTES/MSNPs ratio was ≈6:1 (g/g). After cooling to room temperature, the MSNPs were separated by centrifugation at 4500 RPM for 1 h, washed with toluene, and then with methanol. Finally, the MSNPs were dried at 50°C for 24 h.

### 2.4 Characterization of MSNPs

For the characterization of the produced MSNPs, the total pore volume, surface area, and pore size distribution were measured using N_2_ adsorption–desorption tests (Quantachrome‐ Quadrasorb Evo 4). The specific surface area was determined using the Brunauer–Emmett–Teller (BET) method. The pore size distribution was calculated according to the Density Functional Theory (DFT) model, while the pore volume was determined by the amount of N_2_ adsorbed at ≈0.99 relative pressure. Field‐emission scanning electronic microscopy (ZEISS GeminiSEM 500) was employed to examine the surface and pore morphology. The zeta potential of MSNP was measured in distilled water with Micromeritics‐Nanoplus 3 at 25°C. The crystal structure of MSNP was determined using a Panalytical Empyrean X‐ray diffraction (XRD) device. XRD analysis was conducted with a CuKα source having a wavelength of 1.5405 Å and a scan speed of 1°/min in the range of 10° –90° (2θ). In order to analyze the chemical bonding structures of OnsA, MSNP, and MSNP@OnsA, Fourier transform infrared spectroscopy (FTIR) analysis was conducted.

### 2.5 Loading and Release of Extract into MSNPs

Plant extract‐loaded MSNPs (MSNP@Ons) were dissolved in a 50% ethanol‐deionized water solution, filtered through filter paper, and a 3 mg/mL loading solution was prepared. MSNP, in a 4/3 weight ratio relative to the extract, was added to the prepared solution, and the mixture was stirred continuously at room temperature in a closed container using a magnetic stirrer for 24 h. Afterward, the MSNP was separated from the solution by centrifugation and left to dry at 50°C in an oven for 24 h. The extract‐loaded MSNPs were coded as MSNP@Ons. The extract loading capacity (LC, w/w %) was determined by comparing the mass loss during combustion between MSNP and MSNP@Ons. For thermogravimetric analysis (TG) curves, ≈15 mg of samples were heated under a nitrogen atmosphere using a Setaram‐Labsys Evo device with a heating rate of 5°C/min until 800°C. Extract loading efficiency (LE, concentration/ concentration %) was calculated by using the concentration difference of the extract solution before and after loading. The absorbance of the supernatant was measured at a wavelength of 320 nm (peak point of absorbance spectrum) using a UV–vis spectrophotometer (Agilent, Cary60). The extract LE of MSNP was calculated by the following Equation (1)

(1) $$\mathrm{LE}=\left(\frac{C_{i}‐C_{f}}{C_{i}}\right)\;\times 100$$

where *C*_i_ (mg/mL) is the initial solution concentration and *C*_*f*_ (mg/mL) is the solution concentration after loading.

The extract release performance of MSNP@Ons was determined by incubating them in phosphate buffer solutions at pH = 7.2 and pH = 5.5. 25 mg MSNP@Ons was added to 10 mL of solution, and release experiments were conducted at a constant stirring speed of 300 rpm on a heated magnetic stirrer at 36°C. During the release process, samples (2 mL) were taken at specific intervals (1, 3, 6, 12, 24, 48, 72, and 120 h), and an equal volume of buffer solution was added to maintain the total volume constant. For total phenolic compound analysis, the sample solution (0.25 mL) was mixed with diluted Folin–Ciocalteu reagent (1.0 mL, 1:9) and shaken vigorously. After 3 min, Na_2_CO_3_ solution (0.75 mL, 1.0%) was added, and the absorbance of the sample was measured at 760 nm after incubating at room temperature for 2 h. The total phenolic content was expressed as gallic acid equivalents and evaluated as the release amount [24].

The release profiles obtained were mathematically evaluated using the first‐order kinetic model. The first‐order model assumes that the release rate depends on the amount of remaining drug. In this model, Equation (2) describes the relationship, where *C*_t_ represents the amount of drug released at time *t*, *C*_*max*_ represents the maximum release potential, and *k*_1_ is the release rate constant. *C*_t_ was calculated by the following Equation (2)

(2) $$C_{t}=C_{max}(1‐exp(‐k_{1}.t))$$

### 2.6 Minimum Inhibition Concentration

To determine the Minumum Inhibitory Concentration (MIC) value of the plant extract, the Clinical Laboratory Standards Institute M7‐A8 protocol was modified and applied [27, 28, 29]. For the broth microdilution assay, sterile U‐bottom 96‐well microplates were used. *S. aureus* ATCC 25923 was incubated overnight at 37°C in Mueller Hinton Broth. The bacterial suspension was prepared at a final concentration of 1 × 10^6^ cells/mL. To achieve this, the bacterial liquid culture was diluted 1:100 with fresh sterile medium, and its optical density (OD_590_ nm) was measured using a spectrophotometer [27, 28]. A 100 μL aliquot of the prepared bacterial suspension (1 × 10^6^ cells/mL) was added to each well. Subsequently, 100 μL of plant extract at different concentrations (0.24–1000 μg/mL, in two‐fold serial dilutions) was added to the wells and incubated overnight at 37°C. After incubation, absorbance was measured at 545 nm using a microplate reader. Ciprofloxacin (1 mg/mL, 100 μL) was used as the positive control, while the medium served as the negative control [27]. The MIC value was determined as the lowest concentration at which no visible microbial growth was observed compared to the control groups. The same procedure was followed to determine the MIC values of MSNP and extract‐loaded MSNP. The initial dilution of MSNP and extract‐loaded MSNP was prepared at 1000 mg/mL and tested in eight serial dilutions (1/2, 1/4, 1/8, 1/16, 1/32, 1/64, 1/128, and 1/256) [30]. These dilutions were added to each well in 100 μL volumes, following the same methodology as for the plant extract MIC determination. All analyses were performed in at least three independent replicates.

### 2.7 Determination of the Antibiofilm Effect

3‐(4,5‐dimethylthiazol‐2‐yl)−2,5‐diphenyltetrazolium bromide (MTT) reduction test was used to determine the antibiofilm effect at ½ × MIC, MIC, 2 × MIC concentrations of plant extract, MSNP and extract‐loaded MSNP [31, 32, 33]. *S. aureus* ATCC 25923 was incubated in Tryptic Soy Broth (TSB) medium containing 1% glucose for 24 h at 37°C. 100 μL of bacterial suspension (1 × 10^6^ cells/mL) was added to 96‐well plates. Then 100 μL of each treatment group was added to 96‐well plates and incubated at 37°C for 24 h. 100 μL ciprofloxacin (1 mg/mL) was used as a positive control. Medium was used as negative control [27]. After incubation, the supernatants were discarded and the wells were washed three times with PBS. 150 μL PBS and 50 μL MTT (0.3%) were added and incubated at 37°C for 2 h. MTT solution was removed from the wells and 150 μL DMSO, 25 μL 0.1 M glycine buffer (pH 10.2) was added to the wells to dissolve the formazan crystals and incubated for 15 min at room temperature. The optical density was then measured with a microplate reader at a wavelength of 570 nm. All tests were repeated three times to increase the scientific consistency of the results. The results are given as mean value and standard deviation (mean ± SD). Tukey test was applied to determine the statistical similarities/differences between the data (α = 0.05). To calculate the percentage of biofilm inhibition, the following Equation (3) was used

(3) $$\text{Inhibition }(\%)=\left[1-\left(\frac{A_{570}\;\text{of the test}}{A_{570}\;\text{of none teated control}}\right)\right]\times 100$$

### 2.8 Determination of the Biofilm Eradication

Minimum biofilm eradication concentration was determined to determine the antibiofilm effect of ½ x MIC, MIC, 2 x MIC concentrations of plant extract, MSNP and extract‐loaded MSNP on the biofilm structure formed by *S. aureus* ATCC 25 923 [32]. 200 μL of bacterial suspension (1x10^6^ cells/mL) was transferred to 96‐well plates and incubated at 37°C for 24 h to allow biofilm formation. After biofilm formation, the wells were carefully washed three times with PBS to remove bacterial cells that did not adhere to the wells. Then 200 μL of each treatment group was added to the wells and incubated at 37°C for 24 h. The MTT reduction assay was then performed as described above. 100 μL ciprofloxacin (1 mg/mL) was used as a positive control. The medium was used as a negative control [27]. All tests were repeated three times to increase the scientific consistency of the results. The results are given as mean value and standard deviation (mean ± SD). Tukey test was applied to determine the statistical similarities/differences between the data (α = 0.05). The percentage of eradication was calculated using Equation (4).

(4) $$\text{Biofilm eradiction}(\%)=\left[1-\left(\frac{A_{570}\;\text{of the test}}{A_{570}\;\text{of none teated control}}\right)\right]\times 100$$

### 2.9 Evaluation of Antibiofilm Effect by Microscopic Methods

Antibiofilm effect of plant extract, MSNP and extract loaded MSNP on *S. aureus* ATCC 25923 was determined by scanning electron microscopy (SEM). In a previous study, it was determined that *S. aureus* ATCC 25923 is a strong biofilm producer [31, 34]. For microscopic analyses, the surface area of each piece of positively charged slides was cut to 1 cm^2^ and cleaned with 70% ethanol for 10 min. The blocks will be washed with sterile distilled water and sterilised in an autoclave at 121°C under 1.5 atm pressure for 15 min. These blocks were individually placed in 24‐well plates. Treatment groups were formed with ½ x MIC, MIC, 2 x MIC concentrations of plant extract, MSNP and extract loaded MSNP. 200 μL of these concentrations prepared in TSB was added to each well. 200 μL of bacterial suspension (1x10^6^ cells/mL) was added to each well. Only TSB added medium was used as a positive control for biofilm formation. The plates were incubated at 37°C for 24 h. The samples were then prepared for SEM analysis. SEM analysis was performed within the scope of service procurement. For this purpose, the samples were first placed in a solution containing 2.5% glutaraldehyde (0.1 M phosphate buffer, pH 7.4) at 4°C for 24 h for primary fixation. At the end of the time, the samples were washed 3 times with PBS. They were then placed in osmium tetroxide for 2 h in the dark. After this step, the samples were dehydrated with increasing concentrations of ethyl alcohol (30%, 50%, 70%, 90%, and 96%), dried and coated with gold (Polaron SC7620 Sputter Coater) [35]. All analyses were performed in at least three replicates.

## 3 Results

### 3.1 Total Phenolic Content and the Phytochemical Analysis

*O. armenum* extract was obtained with 14.5% yield. The amounts of total phenolic and flavonolic content in the plant extract were determined and shown in Table 1. The presence and amounts of 30 phytochemicals in the extracts were determined using a previously validated method. The results revealed the presence of many important phytochemicals such as hesperidin, chlorogenic acid, rosmarinic acid, 3‐hydroxybenzoic acid, 4‐hydroxybenzoic acid, 2,5‐dihydroxybenzoic acid, protocatechuic acid, p‐coumaric acid, caffeic acid and hyperoside as major components in the extracts (Table 2). Among these compounds, hesperidin (14201 µg/g extract), chlorogenic acid (4390 µg/g extract) and rosmarinic acid (830 µg/g extract) were detected at higher concentrations than other phytochemicals.

### 3.2 Characterization of MSNP and Preperation of the Plant Extract Loaded MSNP

**FIGURE 1:**
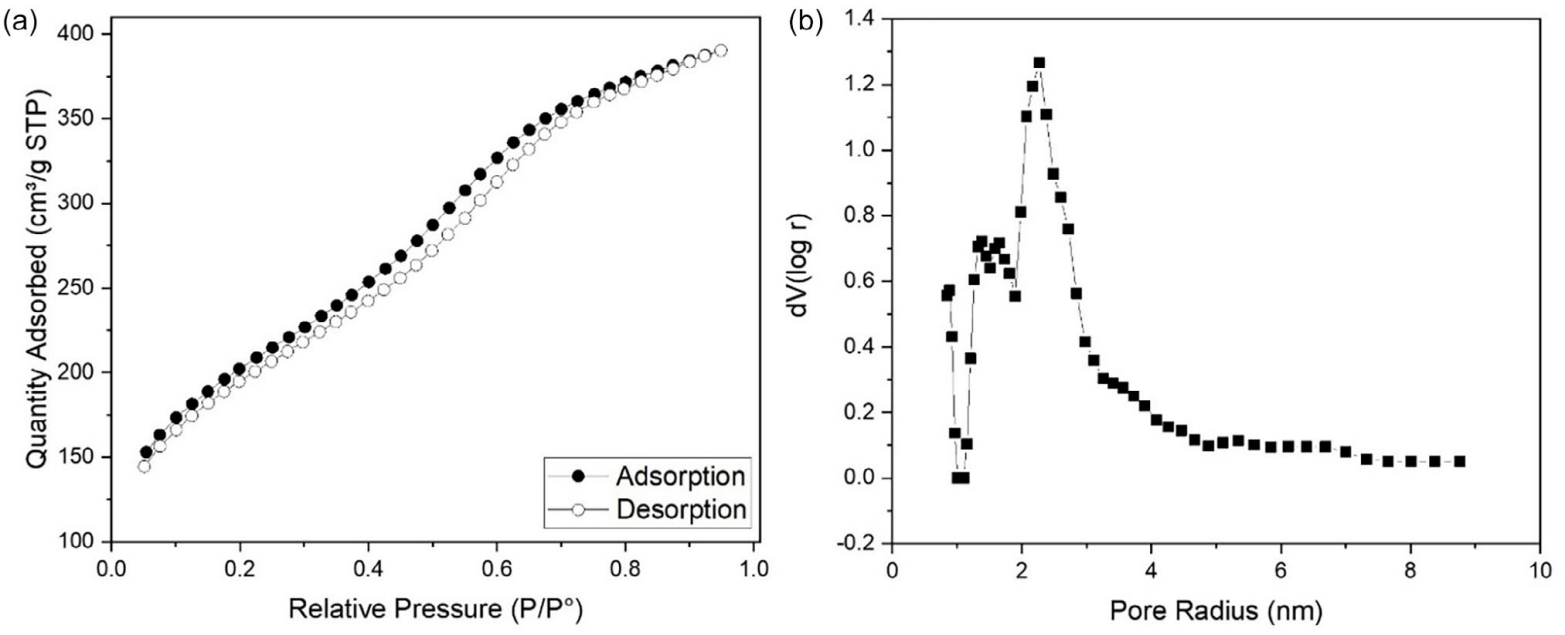
(a) N_2_ adsorption–desorption isotherm of MSNP and (b) pore size distribution graph.

Figure 1 shows the N_2_ adsorption–desorption isotherm and pore size distribution of the MSNP. The pore size distribution is concentrated within the 3 nm range. The specific surface area (BET) of the MSNP was measured as 706 m^2^/g, while the total pore volume was 0.6 cm^3^/g.

**FIGURE 2:**
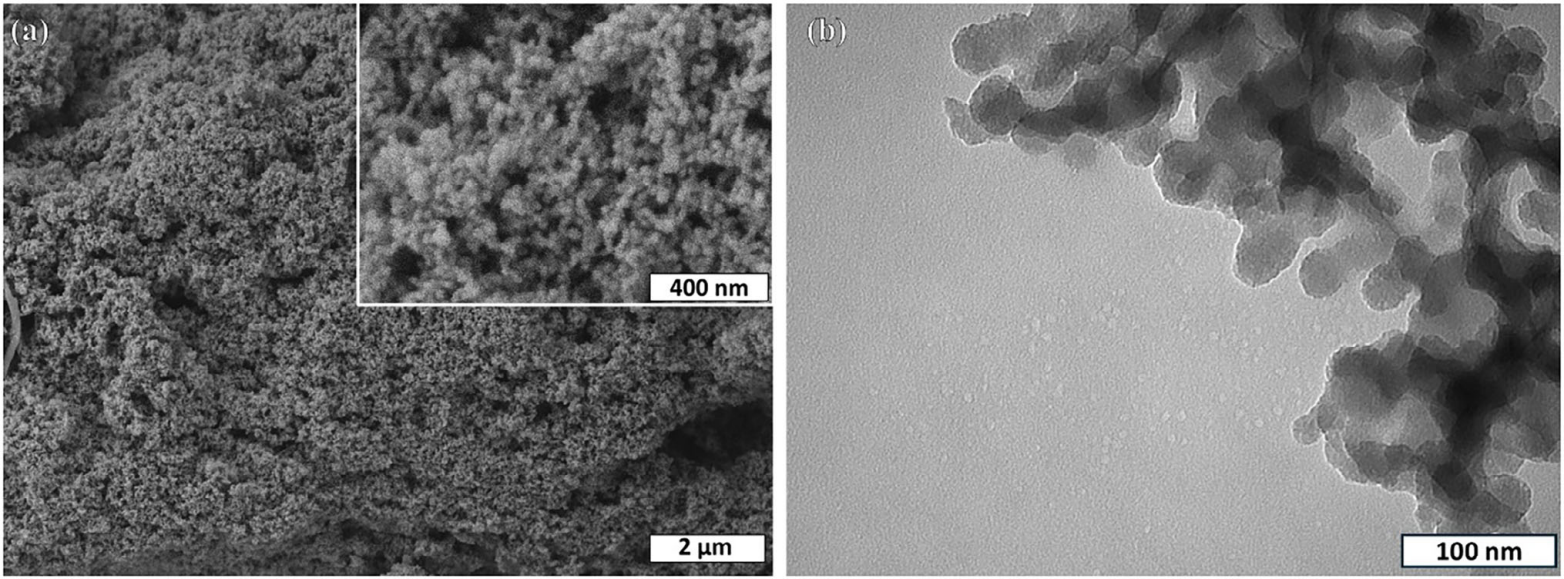
(a) SEM images of MSNP surface at 20,000x and 100,000x magnification (inlet) (b) TEM image of MSNP.

Figure 2a displays SEM images of the surface of the produced MSNPs taken at magnifications of 20,000x and 100,000x. TEM images of MSNPs are shown in Figure 2b.

**FIGURE 3:**
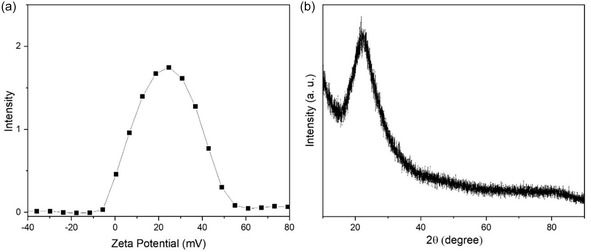
(a) Zeta‐potential graph and (b) XRD diffraction pattern of MSNP.

The zeta potential of MSNPs is presented in Figure 3a. The zeta potential measured in deionized water was ≈ +21 mV, indicating successful surface functionalization. The XRD pattern shown in Figure 3b reveals a hill‐like peak around 21°.

**FIGURE 4:**
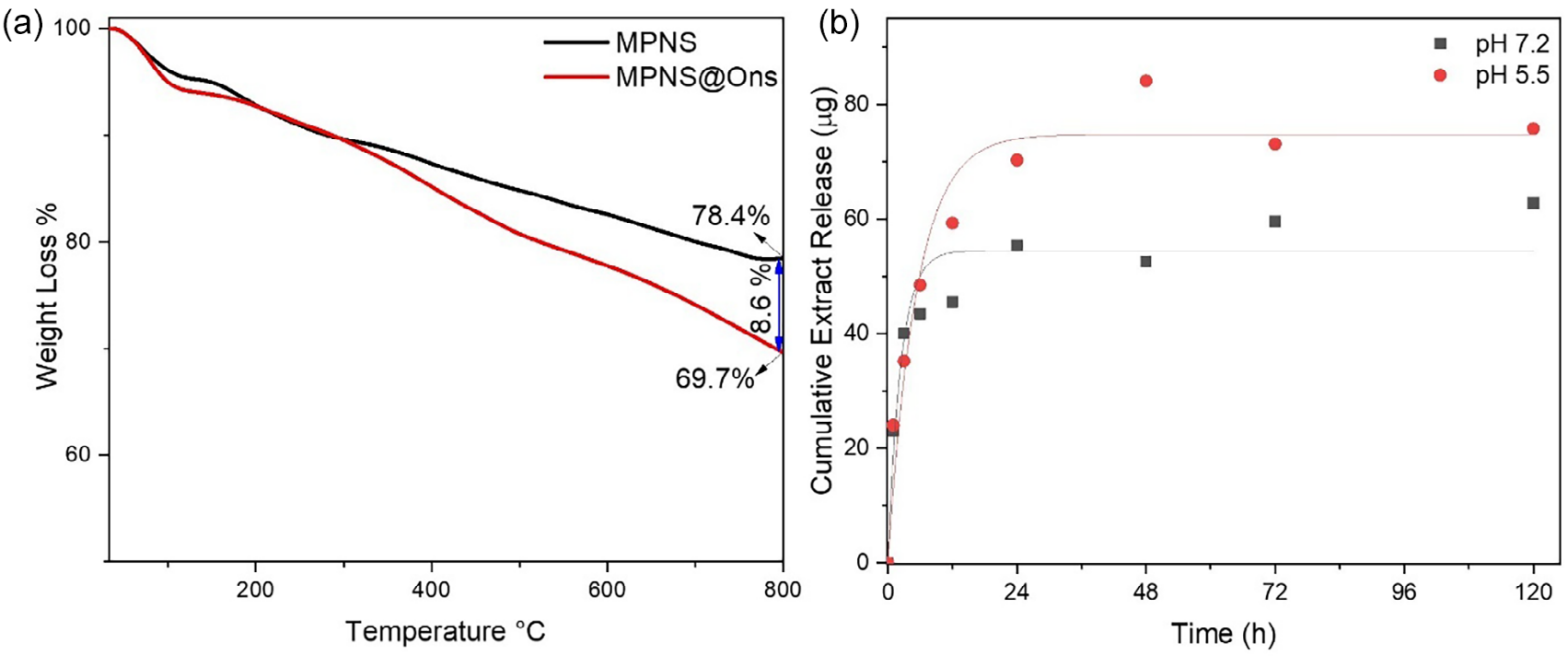
(a) TG curves of MSNP and MSNP@Ons and (b) extract release profiles according to the first‐order kinetic model.

The LC of MSNP was determined by the mass change graph measured by TG. According to the TG curves shown in Figure 4a, the LC of MSNP for *O. armenum* was ≈8.6%. In addition, LE was determined as 28.25%. The analysis results based on the first‐order model are shown in Figure 4b. The *R* ^2^ values for neutral and acidic environments were calculated to be 0.92 and 0.95, respectively, indicating good agreement with the release characteristics.

**FIGURE 5:**
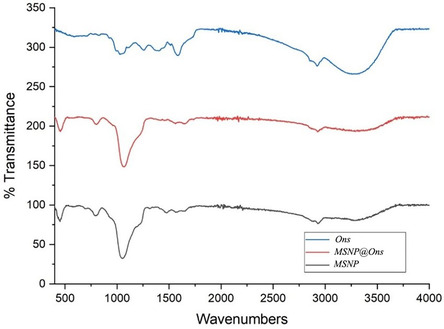
FTIR spectra of Ons, MSNP, and MSNP@Ons.

The FTIR spectra were recorded using a Thermo Scientific Nicolet iS20 device equipped with an attenuated total reflectance accessory, within the wavenumber range of 400–4000 cm^−1^ and at a resolution of 4 cm^−1^. In Figure 5, the sharp and intense peak observed at 1040 cm^−1^ in the FTIR spectrum of Ons corresponds to the C–O stretching vibration of the hydroxymethyl group within the phenolic hydroxyl ring [36, 37, 38]. This peak supports the extract's high total phenolic content and antioxidant capacity. The peak observed around 1260 cm^−1^ is attributed to the C–N stretching vibration of a primary aliphatic amine. The absorption band between 1380 and 1420 cm^−1^ corresponds to the O–H bending vibration of phenolic compounds, further confirming the presence of phenolic groups in the extract. In the region between 1530 and 1630 cm^−1^, a stretching vibration band associated with secondary amide N–C=O groups is detected. The peak at 1730 cm^−1^ is related to the carbonyl (C = O) functional group [38, 39]. The broad band observed between 3030 and 3600 cm^−1^ indicates the presence of inter‐ and intramolecular hydrogen bonding, corresponding to O–H stretching vibrations.

In the FTIR spectra of MSNP and MSNP@Ons shown in Figure 5, a broad peak around 1050 cm^−1^ is prominently observed, which is attributed to the presence of SiO_2_ in MSNP [38, 40]. Additionally, characteristic Si–O–Si stretching vibrations are evident at 801 and 792 cm^−1^ in the FTIR spectra of MSNP and MSNP@Ons, respectively. When comparing the FTIR spectra of OnsA and MSNP@Ons, it is considered that some of the functional groups associated with the phytochemicals in the plant extract are not clearly observed in the MSNP@Ons spectrum. This may be due to the overlapping and dominance of the broad SiO_2_ peak in that region.

### 3.3 Determination of Antimicrobial Effect

**FIGURE 6:**
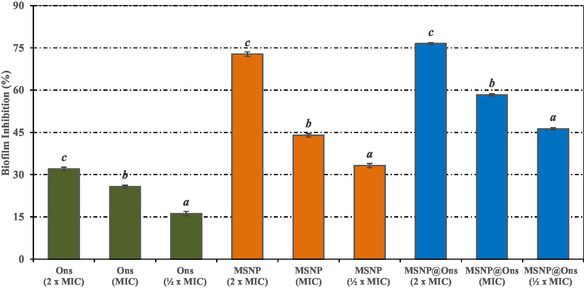
Antibiofilm effect of treatment groups on *S. aureus* ATCC 25923. Values are expressed as mean ± SD (*n* = 3). Statistical differences were evaluated by one‐way analysis of variance (ANOVA) followed by Tukey's honestly significant difference (HSD) post hoc test. Values sharing the same superscript letter are not significantly different at *p* < 0.05.

The minimum inhibitory concentration of *O. armenum* extract against *S. aureus* ATCC 25923 was determined to be 250 µg/mL. The MTT reduction assay was used to evaluate the antibiofilm effects of Ons, MSNP, and MSNP@Ons at ½ × MIC, MIC, and 2 × MIC concentrations against *S. aureus* ATCC 25923. It was observed that the percentage of biofilm inhibition increased proportionally with the concentration of Ons, MSNP, and MSNP@Ons. The antibiofilm effects of different concentrations of the plant extract on *S. aureus* ATCC 25923 are shown in Figure 6. Biofilm inhibition rates of 31.97 ± 0.68%, 72.78 ± 0.79%, and 76.49 ± 0.39% were observed in the groups treated with 2 × MIC concentrations of Ons, MSNP, and MSNP@Ons, respectively.

**FIGURE 7:**
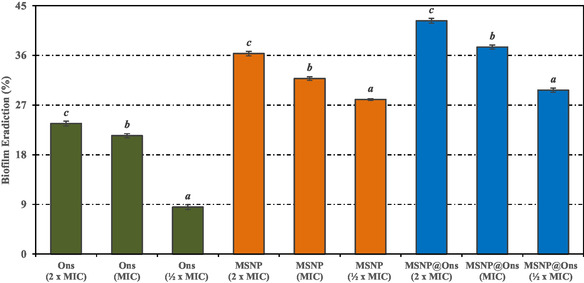
Effect of treatment groups on biofilm eradication on *S. aureus* ATCC 25923. Values are expressed as mean ± SD (*n* = 3). Statistical differences were evaluated by one‐way ANOVA followed by Tukey's HSD post hoc test. Values sharing the same superscript letter are not significantly different at *p* < 0.05.

The biofilm eradication effects of Ons, MSNP, and MSNP@Ons at ½ × MIC, MIC, and 2 × MIC concentrations on the biofilm structure formed by *S. aureus* ATCC 25 923 were determined and are shown in Figure 7. It was observed that the percentage of biofilm eradication increased proportionally with the concentration of Ons, MSNP, and MSNP@Ons. In the groups treated with 2 × MIC concentrations, biofilm eradication rates of 23.66 ± 0.44%, 36.33 ± 0.40%, and 42.27 ± 0.44% were observed for Ons, MSNP, and MSNP@Ons, respectively.

**FIGURE 8:**
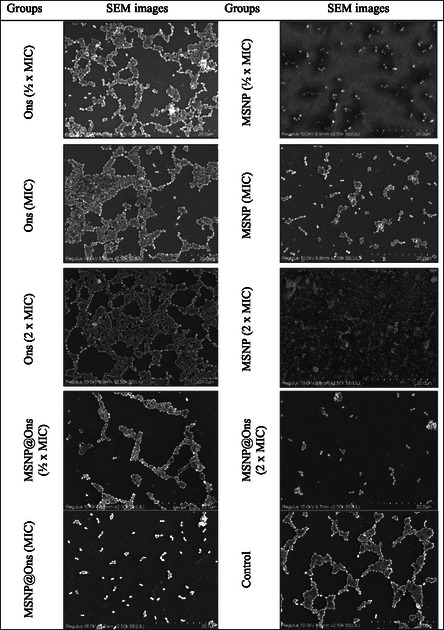
SEM images of antibiofilm effect of treatment groups on *S. aureus* ATCC 25923.

Additionally, the antibiofilm effects of Ons, MSNP, and MSNP@Ons on *S. aureus* ATCC 25923 were determined using SEM analysis. The SEM analysis results were consistent with the microbiological findings, demonstrating that biofilm inhibition increased proportionally with the concentration of Ons, MSNP, and MSNP@Ons (Figure 8). According to SEM analysis, in the group treated with ½ × MIC Ons, although the cellular structures were generally preserved, some morphological deformations were observed. Cellular fusions were detected, but no significant reduction in cell numbers was noted compared to the control group. In the MIC Ons‐treated group, while no noticeable decrease in cell count was observed, cellular damage was evident. Some cells exhibited swelling, lysis, cellular fusions, shrunken structures, and bleb formations. In the 2 × MIC Ons‐treated group, similar morphological deformations were observed as in the MIC Ons‐treated group.

In the ½ × MIC MSNP‐treated group, a reduction in cell numbers was observed compared to the control group. Cells appeared smaller with atypical morphological alterations, indicating biofilm degradation. In the MIC MSNP‐treated group, a similar reduction in cell numbers was observed, along with cellular damage due to MSNP interaction. Lysis of cells and leakage of intracellular content were detected, as well as cellular fusions. Biofilm degradation was evident. In the 2 × MIC MSNP‐treated group, a further reduction in cell numbers was noted compared to the control group. Significant biofilm degradation was detected, with extensive cellular damage and fragmentation of microorganisms. In the ½ × MIC MSNP@Ons‐treated group, a reduction in cell numbers was observed compared to the control group. Cellular structures were disrupted, cellular fusions were evident, and cells were fragmented. In the MIC MSNP@Ons‐treated group, a further reduction in cell numbers was observed, with a high degree of antibiofilm activity. The biofilm structure was disrupted into fragments. Nanoparticles were found to accumulate on bacterial surfaces, leading to cellular damage. In the 2 × MIC MSNP@Ons‐treated group, a significant reduction in cell numbers was observed compared to the control group. A strong antibiofilm effect was detected, with bacterial disintegration likely caused by the impact on the cell wall.

## 4 Discussions

Medicinal and aromatic plants have been used for centuries in the treatment of diseases due to their bioactive components. Natural phenolic compounds in these plants are known for their beneficial effects, such as antioxidant, antiaging, anti‐inflammatory, and antimicrobial activities. The growing concern over the side effects of synthetic drugs has increased interest in exploring and developing natural alternatives derived from these plants. Clinically, most chronic infections are linked to bacterial biofilms, which exhibit high resistance to antibiotics and can persist even under unfavorable conditions [41]. Biofilm‐associated infections pose a significant clinical challenge, as they can form on both medical devices and biological surfaces, leading to increased patient morbidity and mortality. The widespread prevalence of multidrug‐resistant bacteria further exacerbates this issue, making biofilm infections a major threat, particularly in hospitalized patients. Although numerous studies have explored biofilm formation on various surfaces, research has largely been limited to specific materials and bacterial species. Therefore, a deeper focus on bacterial biofilm infections is essential for developing more effective prevention and treatment strategies [6, 42].

Recent studies report that the therapeutic effects of plants are not due to a single active compound but rather the synergistic effects of multiple components, which can provide more effective treatment by counteracting the resistance of microorganisms that are difficult to kill with a single antibiotic [43]. This has led researchers to explore natural antimicrobial agents derived from plant extracts that have inhibitory effects [44]. Antimicrobial activity is influenced by factors such as the plant species, composition, and concentration, the type and load of the target microorganism, the composition of food, and processing and storage conditions. Proteins, lipids, salts, pH, and temperature are factors that affect the antimicrobial activities of phenolic compounds [45]. Research in this area has shown that plant materials contain a wide range of phytochemical compounds with strong antioxidant and antimicrobial activities [46].

Nanomaterials offer a promising strategy for combating bacterial biofilms by reducing their adhesion, enhancing the delivery and permeability of antimicrobial agents, and maintaining antibiotic stability. Notably, they can also exhibit biofilm resistance through specific mechanisms independent of antibiotics. Due to their unique physical and chemical properties, including biological response, surface charge, and nanoscale effects, nanomaterials can engage in multiple antibacterial interactions with bacterial cells. These materials serve as effective carriers for antimicrobial agents, inhibiting biofilm growth through mechanisms such as thermal damage, oxidative stress, and physical disruption, thereby aiding in the treatment of biofilm‐associated infections [47]. Additionally, their mode of action makes them less prone to inducing drug resistance [48]. Nanomaterials enhance antibacterial efficacy through several mechanisms. Their surface charge facilitates interactions with bacterial cell membranes, extending antibiotic exposure. Some nanomaterials prevent bacterial resistance by employing self‐cracking mechanisms, while others improve the solubility, stability, and targeted release of antimicrobial agents. Furthermore, they enable combination therapies, such as photothermal and photodynamic treatments, by delivering multiple agents to the same target site. Certain nanovesicles also prevent premature drug degradation and enable precise drug delivery through membrane fusion [49]. Given these advantages, nanomaterials present a compelling avenue for addressing biofilm‐related infections and overcoming the challenges posed by antibiotic resistance. Nanoparticle‐based therapeutic strategies hold significant potential for effectively eliminating bacterial biofilms due to their unique advantages, including functional versatility, selectivity, traceability, high drug‐LC, and controlled release [50, 51]. Unlike conventional antimicrobials, activated nanoparticles can be engineered to respond to specific triggers, minimizing off‐target effects and reducing potential side effects [52]. Given these advantages, the continued development of nanomaterials for treating biofilm‐associated infections remains a crucial area of research with promising clinical implications [53, 54, 55].

In this study, the phenolic content of *O. armenum* extract was identified, and MSNPs were synthesized and characterized. This is the first study to investigate the potential use of *O. armenum* extract‐loaded MSNP in the treatment of biofilm‐associated infections, utilizing microbiological, microscopic, and molecular methods. The findings suggest that the combination of the extract and MSNPs exhibits enhanced antimicrobial and antibiofilm activities compared to the individual components.

The shape of N_2_ adsorpton–desorption isotherm is determined by the pore structure, and according to IUPAC classifications, MSNP exhibit a type IV isotherm, with a hysteresis loop, which is commonly associated with mesoporous materials [56]. The characteristic structure of mesoporous silica is observed in the produced samples, and the mesoporous structure is maintained after the functionalization process. The obtained porosity is similar to that of mesoporous silicas produced by similar methods in literature and at a satisfactory level for drug release applications [57, 58, 59].

The SEM images reveal that the characteristic mesoporous structure of silica was clearly observed in the synthesized samples and was preserved following the functionalization process TEM images show that MSNP consists of spherical particles with an average diameter of 25 nm. In agreement with previous studies, a nanoporous architecture was evident, characterized by small spherical aggregates forming an interconnected porous framework [60]. Mesoporous silicas typically exhibit a negative surface charge due to the presence of silanol (Si–OH) groups on their surface [61]. However, after surface modification with positively charged ‐NH_2_ groups, the zeta potential shifted to positive values. A zeta potential of + 21 mV indicates a stable surface charge, which is crucial for enhancing the interaction of the nanoparticles with bioactive compounds or cells.

The XRD pattern further confirms the characteristic amorphous silica, as evidenced by the distinctive broad peak centered at 21°, typical of amorphous silicas [62]. The LC and LE of MSNPs for *O. armenum* extract were found to be 8.6% and 28.25%, respectively, which are in line with values reported in the literature [63, 64]. The drug release profiles, as analyzed using the first‐order kinetic model, showed strong agreement with the experimental data, with *R* ^2^ values of 0.92 and 0.95 for neutral and acidic conditions, respectively. The higher total release capacity in acidic conditions (74.80 μg) and the slower release rate (0.18 μg/hour) suggest that the MSNPs are capable of prolonged, controlled release, particularly in environments with lower pH, which is beneficial for controlled drug delivery. This pH‐responsive release behavior can be attributed to the protonation of ‐NH_2_ groups on the MSNP surface under acidic conditions, as confirmed by previous studies [65, 66].

The extract of *O. armenum* was found to contain 30 phytochemicals, with significant concentrations of hesperidin (14201 µg/g extract), chlorogenic acid (4390 µg/g extract), and rosmarinic acid (830 µg/g extract) when compared to other phytochemicals (Table 1). These findings align with previous research, such as the study by Jabbar et al. [67], which highlighted the extensive ethnopharmacological applications of *Onosma* species, including their antioxidant, antimicrobial, anti‐inflammatory, and anticancer properties. The high concentration of hesperidin in *O. armenum* extract is particularly noteworthy, as this flavonoid glycoside has been shown to possess diverse pharmacological activities, including antimicrobial, antioxidant, and anticancer properties. Its ability to disrupt bacterial cell walls and induce oxidative stress leading to macromolecular leakage suggests that hesperidin may be an important contributor to the antimicrobial efficacy observed in this study.

The comparative study by Panou et al. [68] on *Onosma* species’ phenolic profiles further supports the potential of *O. armenum* in therapeutic applications. Similar to other *Onosma* species, *O. armenum* contains high levels of bioactive compounds such as chlorogenic acid, a compound known for its potent antioxidant and antimicrobial activities [25, 69]. Chlorogenic acid's role in lipid metabolism and glucose regulation highlights its therapeutic potential in addressing metabolic disorders, further supporting the relevance of *O. armenum* as a source of natural bioactive agents. Another study by Sarikurkcu and and Tlili [20] explored the phytochemical content of *Onosma inexspectata* Teppner and *O. armenum* DC. The phenolic compound levels for *O. inexspectata* and *O. armenum* were found to be ≈22 and 28 mg gallic acid equivalent/g, respectively. A total of twenty‐seven compounds were identified and quantified through LC‐ESI‐MS/MS. The major compounds found in *O. inexspectata* included chlorogenic acid, apigenin 7‐glucoside, and luteolin 7‐glucoside, while *O. armenum* was found to contain considerable amounts of hesperidin.

Hesperidin, a flavonoid glycoside, is recognized for its various pharmacological activities, including antibacterial, antioxidant, and anticancer properties. It can damage bacterial cell walls and induce the leakage of bacterial macromolecules by generating reactive oxygen species. Furthermore, hesperidin has been shown to improve motor dysfunction related to neuropathological degeneration and spinal cord injury through its antioxidant and anti‐inflammatory mechanisms [70]. Similarly, chlorogenic acid, known for its antioxidant, antimicrobial, and hepatoprotective properties, has shown promise in addressing metabolic disorders and promoting lipid and glucose metabolism [69]. Rosmarinic acid, a naturally occurring phenolic compound found in many plants, is known for its antioxidant, anti‐inflammatory, anticancer, and antimicrobial activities. Its derivatives, such as salvianolic acid and lithospermic acid, play important roles in plant defense mechanisms and have demonstrated significant therapeutic potential in a variety of pharmacological applications [71].

The results of this study demonstrate that MSNP@Ons exhibited a significantly higher antibiofilm activity compared to the extract alone or the nanoparticles without the extract. The highest antibiofilm activity was determined in the MSNP@Ons group (76.49 ± 0.39%). This enhancement can be attributed to the synergistic effect between the extract's bioactive components and MSNPs. SEM analysis confirmed that the nanoparticles, both with and without the extract, clustered on the bacterial surface, suggesting a direct interaction that may disrupt the bacterial cell wall structure. The SEM images further illustrated the damage to the structural integrity of bacterial cells, leading to cell shrinkage and the formation of gaps, consistent with the increasing concentrations of the nanoparticles and extract.

The results clearly demonstrate that MSNP@Ons exhibits superior antibiofilm activity compared to both the free *O. armenum* extract and bare MSNPs. This enhanced efficacy can be attributed to a synergistic effect arising from the integration of the bioactive plant extract with the MSNP carrier. Encapsulation within MSNPs likely improves the stability of phenolic constituents, facilitates controlled and sustained release, and enhances surface interaction with bacterial biofilms. In contrast, the free extract may suffer from limited stability and rapid diffusion, while bare MSNPs lack intrinsic antibiofilm activity. Therefore, the markedly improved performance of MSNP@Ons highlights the advantage of nanoparticle‐based delivery systems in maximizing the antibiofilm potential of plant‐derived bioactive compounds. These findings further suggest that MSNPs do not merely act as passive carriers but also play an active role in enhancing the antibiofilm efficacy of *O. armenum* extract through synergistic physicochemical interactions.

Studies conducted to date have revealed that phenolic compounds possess significant antibiofilm activity [72]. Apigenin, a phenolic compound, inhibited biofilm formation of *Streptococcus sobrinus* [73]. Gallic acid reduced *S. aureus* biofilm formation by ≈40% at 4 mg/mL and even affected the metabolic activity of attached cells at lower concentrations, surpassing the effects observed in the positive control group. Similarly, verbascoside, used as an antibacterial additive in fresh meat storage, effectively killed bacteria at medium and high doses, reducing spoilage rates and extending the meat's shelf life [74, 75]. Chlorogenic acid significantly inhibited biofilm formation by *Yersinia enterocolitica* in a dose‐dependent manner, effectively reducing the biofilm layer [74]. In another study, Shilov et al. [76] examined the composition and biological activity of various extracts derived from the roots of *Onosma gmelinii*. The presence of shikonin and its derivatives was verified in all the extracts. The concentration of naphthoquinones was found to be ≈40% in the CO_2_ extract, 3% in the ultrasonic extract, and 1.3% in the percolation extract. The biological activity of these extracts was assessed against several bacteria, viruses, and fungi species. The root extracts of *Onosma gmelinii* exhibited varying degrees of antibacterial activity against all tested gram‐positive bacterial strains, while no inhibitory effects were observed on gram‐negative bacteria. The results showed that the ultrasonic extract effectively inhibited the growth of most tested gram‐positive bacteria (MBC 18.3–293.0 µg/mL). The CO_2_ extract exhibited the highest bactericidal activity, while the percolation extract had little effect on bacterial growth. The CO_2_ and ultrasonic extracts significantly reduced the activity of *C. albicans*. The antiviral activity results indicated that the ultrasonic extract had the highest efficacy against different subtypes of influenza virus, and the CO_2_ extract exhibited moderate antiviral activity.

In another study, Kostić et al. [77] aimed to phytochemically characterize two endemic Balkan species, *Onosma stellulata* and determine their potential applications as natural antioxidant and antimicrobial agents. The main phenolic compound identified was chlorogenic acid. Additionally, isorhamnetin‐3‐O‐rutinoside and sinapic acid were also detected in *O. stellulata*. Antimicrobial tests showed moderate antibacterial activity, particularly against several fungi, with notable inhibition against *Trichoderma viride*. Correlation analysis revealed a strong positive link between phenolic compounds and the reduction power of the extracts, as well as a strong connection between total phenolic and flavonoid content and the minimum inhibitory concentration recorded in antibacterial experiments.

Similar to our study, Memar et al. [78] investigated the physicochemical characteristics, anticancer, and antibacterial activities of rutin‐loaded MSNPs, supporting the findings of our research. They reported that the anticancer and antibacterial effects of rutin were significantly enhanced when delivered via MSNPs compared to free rutin. Specifically, the rutin‐loaded MSNPs demonstrated a strong inhibitory effect on the growth of HN5 cancer cells through mechanisms involving ROS generation and apoptosis induction. Notably, their results showed that the rutin‐loaded MSNPs exhibited up to 50 times greater potency in inhibiting HN5 cell proliferation compared to free rutin, emphasizing their potential as adjuvants in cancer therapy. However, further research is necessary to validate and expand upon these findings. In addition to their anticancer potential, rutin‐loaded MSNPs also exhibited superior antibacterial activity relative to free rutin. These results highlight the promise of nanotechnology‐based strategies in enhancing the therapeutic efficacy of natural compounds like rutin, particularly by overcoming challenges such as low bioavailability and limited biological activity.

In a related study, Petrişor et al. [79] explored the antimicrobial potential of MSNPs loaded with *Melissa officinalis* extract. Their findings revealed that the natural hydroalcoholic extract, rich in rosmarinic acid (1286 mg per 100 g extract as quantified by HPLC), could be effectively incorporated into mesoporous silica structures and exhibited notable antimicrobial activity. The formulated nanoparticles demonstrated high efficacy particularly against Gram‐positive bacteria and fungal strains. Among the tested microorganisms, *S. aureus* ATCC 25923 showed the highest sensitivity, with MIC values ranging from 0.156 to 1.25 mg/mL. This was followed by *Candida albicans* ATCC 10 231 (0.078–2.5 mg/mL) and *Enterococcus faecium* ATCC 13048 (0.5 mg/mL). Importantly, the study included both standard and clinical strains, such as methicillin‐resistant *S. aureus* (MRSA) isolated from nosocomial infections, underscoring the potential of *M. officinalis*‐loaded nanoparticles as a promising alternative for managing resistant pathogens. These results support the growing body of evidence that plant‐based compounds, when delivered via nanocarriers, can enhance antimicrobial efficacy and broaden the therapeutic scope against clinically significant microorganisms.

Therefore, the integration of nanotechnology with natural plant extracts represents an innovative and promising strategy for the development of effective antimicrobial systems. Understanding the interaction between the bioactive compounds present in *O. armenum* and the structural and functional properties of MSNPs is crucial for enhancing the design and performance of such nanosystems. This synergy not only contributes to increased antimicrobial efficacy but also supports the broader application of nanotechnology in combating resistant pathogens. As demonstrated in our study, the combination of natural phytochemicals with nanocarriers can overcome limitations such as poor solubility and bioavailability, offering a powerful approach in the advancement of antimicrobial research.

## 5 Conclusions

In conclusion, this study highlights the potential of *O. armenum* extract‐loaded MSNPs as a novel antimicrobial and antibiofilm agent. The high concentrations of bioactive phenolic compounds, particularly hesperidin, chlorogenic acid, and rosmarinic acid, suggest that *O. armenum* may be a valuable resource for developing natural alternatives to combat biofilm‐associated infections. The synergistic effect of the extract and nanoparticles in enhancing antimicrobial and antibiofilm activities provides a strong foundation for further research in the field of medicinal and aromatic plants, particularly those rich in phenolic compounds, as solutions to antibiotic resistance. This study highlights the synergistic effects of phenolic compounds derived from medicinal and aromatic plants against antibiotic resistance and provides a strong foundation for future research in this field. Future research should focus on optimizing the encapsulation and release mechanisms of *O. armenum* extract‐loaded MSNPs and further explore their clinical applications. Additionally, comprehensive in vivo studies are necessary to evaluate the safety, efficacy, and long‐term benefits of these formulations in treating biofilm‐related infections.

## Supporting Information

Additional supporting information can be found online in the Supporting Information section. **Supporting Table S1**: ESI–MS/MS Parameters and analytical characteristics for the Analysis of Target Analytes by MRM Negative and Positive Ionization Mode. **Supporting Table S2**: Calibration curves and sensitivity properties of the method.

## Funding

This study was supported by TÜSEB (33972).

## Conflicts of Interest

The authors declare no conflicts of interest.

## Supporting information

Supplementary Material

## Data Availability

The data that support the findings of this study are available in the supplementary material of this article.
